# Glycogen metabolic dysfunction in T2DM with MASLD: linking α-hydroxybutyrate to GYS2 downregulation

**DOI:** 10.3389/fnut.2026.1860017

**Published:** 2026-06-22

**Authors:** Zichen Zhang, Nuo Chen, Xiaojing Yuan, Jie Li, Wan Zhou

**Affiliations:** 1 Division of Life Sciences and Medicine, Department of Endocrinology and Metabolism, The First Affiliated Hospital of USTC, University of Science and Technology of China, Hefei, China; 2 Division of Life Sciences and Medicine, University of Science and Technology of China, Hefei, China; 3 Department of Endocrinology, The People’s Hospital of Tongcheng, Tongcheng, Anhui, China

**Keywords:** diabetes mellitus, GYS2, metabolic dysfunction-associated steatotic liver disease, metabolomics, RNA-Seq, *α*-hydroxybutyrate

## Abstract

**Objective:**

This study aimed to investigate the molecular characteristics of T2DM with MASLD in a C57 mouse model, using multi-omics techniques to identify key nutritional metabolites that drive metabolic dysfunction. The goal was to reveal how metabolic disturbances are linked to transcriptional reprogramming, providing a new theoretical basis for nutritional biomarker discovery and targeted metabolic interventions.

**Methods:**

In this study, a mouse model of T2DM with MASLD was established using a high-fat diet (HFD) combined with intraperitoneal injection of low-dose streptozotocin (STZ). The mice were randomly allocated into three groups: a healthy control group, a MASLD-only group (HFD), and a T2DM with MASLD group (HFD + 40 mg/kg STZ, ip). Serum untargeted metabolomic analysis and hepatic transcriptomic sequencing were performed to identify significantly differential metabolites and transcripts between groups, specifically focusing on *α*-hydroxybutyrate (α-HB) and glycogen synthase 2 (GYS2). Multi-omics data integration was conducted via Kyoto Encyclopedia of Genes and Genomes (KEGG) pathway enrichment analysis and Pearson correlation analysis. For the validation experiment, both normal mice and T2DM with MASLD mice were further divided into two subgroups, with one subgroup from each cohort receiving *α*-HB intervention to investigate its effects on GYS2 expression and the progression of T2DM with MASLD.

**Results:**

Metabolomic and transcriptomic analyses revealed that *α*-HB levels were significantly elevated in T2DM with MASLD mice, and this elevation showed a significant correlation with the downregulation of GYS2. Validation experiments demonstrated that, compared with normal mice, the T2DM with MASLD mice induced by a high-fat diet combined with STZ injection exhibited significantly increased fasting blood glucose (13.14 ± 0.44 vs. 5.08 ± 0.30 mmol/L, *p* < 0.0001), insulin levels (47.41 ± 1.38 vs. 12.38 ± 1.29 μU/mL, *p* < 0.0001), serum triglycerides (4.75 ± 0.13 vs. 1.27 ± 0.13 mmol/L, *p* < 0.0001), and hepatic triglycerides (61.97 ± 3.53 vs. 17.13 ± 1.01 mg/g, *p* < 0.0001), along with elevated inflammatory factors. GYS2 was significantly downregulated in T2DM with MASLD mice. Compared with saline-treated T2DM with MASLD mice, *α*-HB-treated T2DM with MASLD mice showed a further downregulation of hepatic GYS2 expression (*p* = 0.0011). Following α-HB intervention, T2DM with MASLD mice showed a further downregulation of hepatic GYS2 expression compared to saline-treated T2DM with MASLD mice (*p* = 0.0011). This was accompanied by increased fasting blood glucose (14.70 ± 0.37 vs. 13.14 ± 0.44 mmol/L, *p* = 0.0230) and insulin levels (53.16 ± 0.90 vs. 47.41 ± 1.38 μU/mL, *p* = 0.0086), as well as reduced hepatic glycogen synthesis (21.60 ± 0.97 vs. 35.34 ± 1.40 mg/mg, *p* < 0.0001). Concurrently, serum triglycerides (5.63 ± 0.50 vs. 4.75 ± 0.13 mmol/L, *p* = 0.0160), hepatic triglycerides (70.53 ± 0.69 vs. 61.97 ± 3.53 mg/g, *p* < 0.0398), and the pro-inflammatory cytokines tumor necrosis factor-*α* (TNF-α) (9.69 ± 0.62 vs. 45.70 ± 2.50 ng/L, *p* = 0.0013) and interleukin-6 (IL-6) (55.87 ± 5.39 vs. 37.68 ± 0.78 ng/L, *p* = 0.0029) were all significantly elevated.

**Conclusion:**

This study reveals that *α*-HB, a metabolite elevated under conditions of nutrient overload and insulin resistance, plays an important role in the progression of T2DM with MASLD. It may contribute to a vicious cycle by impairing hepatic energy storage and flux, likely through affecting the activity and expression of GYS2. This finding provides a nutritional metabolism perspective for understanding the T2DM with MASLD comorbidity and suggests *α*-HB as a potential target for nutritional strategies or precision nutrition approaches.

## 1 Introduction

Diabetes mellitus (DM) is a major global health challenge (1, 2), affecting over 10.5% of the adult population worldwide (3). Meanwhile, metabolic dysfunction-associated steatotic liver disease (MASLD) also represents a rapidly growing public health concern, impacting approximately 25% of the global population (4). These metabolic disorders are deeply interconnected, approximately 90% of DM cases classified as type 2 diabetes (T2DM) (5), and 55.5% of T2DM patients also having MASLD (6). Shared underlying factors such as obesity, insulin resistance (IR), and chronic inflammation contribute to their co-occurrence (7). Mechanistically, ectopic fat deposition in the liver disrupts gluconeogenic regulation and adipokine secretion (8, 9), while hyperinsulinemia promotes lipogenesis and mitochondrial dysfunction (10, 11). This creates a vicious cycle where the severity of MASLD is linked to an increased risk of developing T2DM (12, 13) and T2DM accelerates MASLD progression to advanced fibrosis, hepatocellular carcinoma, and cardiovascular complications, establishing a bidirectional risk relationship (14). Particularly in elderly patients, DM with MASLD not only substantially elevates all-cause and cardiovascular-related mortality rates but also severely compromises quality of life and longevity (15). In fact, dietary patterns and nutrient intake are fundamental environmental drivers of these conditions. Chronic overnutrition, particularly diets high in saturated fats and refined carbohydrates, fuels obesity, insulin resistance, and the ectopic lipid accumulation that characterizes MASLD, while simultaneously impairing glycemic control in T2DM. The liver, as the central organ for nutrient processing, becomes a key site where dysregulated nutrient metabolism converges to exacerbate both diseases. Despite the clear epidemiological and pathophysiological associations, the specific molecular pathways that link T2DM and MASLD are not yet fully understood.

The rising global prevalence of these conditions, along with their complex mechanisms, places a significant burden on healthcare systems (14), emphasizing the urgent need to identify common therapeutic targets that can address their intertwined pathophysiology. Advances in bioinformatics now enable novel approaches to addressing these complex scientific challenges, empowering deeper exploration of pathophysiological mechanisms in T2DM with MASLD (16, 17).

Our study employed metabolomics and transcriptomics techniques. Metabolomics analysis focuses on low-molecular-weight metabolites (18), to decipher the specific metabolic links between T2DM and MASLD, a focus on the nutritional metabolome is essential. For T2DM with MASLD, identifying differential nutritional metabolites can reveal early metabolic shifts that precede clinical symptoms, offering insights for nutritional assessment and early intervention (19). Concurrently, metabolomics effectively evaluates therapeutic efficacy in T2DM with MASLD patients by detecting changes in human metabolites, providing more direct insights into drug actions and pathways (20). Transcriptomics investigates all transcripts within biological systems, and understanding transcriptomes is crucial for elucidating genetic determinants of disease progression. Through transcriptome analysis, screening differentially expressed genes reveals potential therapeutic targets for T2DM with MASLD at the genetic level (21, 22).

In the metabolomic analysis, we identified *α*-hydroxybutyrate (α-HB) as a significantly upregulated differential metabolite in the serum of T2DM with MASLD mice. Recent research indicates that α-HB, an exercise-responsive metabolite central to metabolic co-regulation and an early biomarker of insulin resistance (23), may play important roles in insulin resistance, hepatic lipid metabolism dysregulation, and glycogen synthesis regulation (24, 25). However, its specific mechanistic role in the co-morbid progression of T2DM and MASLD remains unclear. Therefore, this study focuses on *α*-HB as a key metabolic hub and further integrates transcriptomic data, aiming to systematically elucidate its molecular regulatory network in the progression of T2DM with MASLD, and ultimately provides new perspectives for developing nutrition-focused therapeutic strategies against T2DM with MASLD.

## 2 Materials and methods

### 2.1 Animals

Our study received approval from the Ethics Committee of The First Affiliated Hospital of USTC and the Medical Institution Animal Care and Research Advisory Committee, following ARRIVE Guidelines (NO.2024-N(A)-0172). The C57BL/6J mice (19–22 g) were purchased from Gempharmatech Co., Ltd. (Nanjing, China). The mice were housed in specific pathogen free conditions with a 12-h light/dark cycle and given free access to food and water. At the experimental endpoint, mice were anesthetized via intraperitoneal injection of a 0.5% pentobarbital solution, followed by euthanasia via cervical dislocation.

### 2.2 Experimental design

1 Bioinformatics analysis experiment

After 1 week of acclimatization, 18 mice were randomly divided into 3 groups (n = 6 per group) as follows:

CON group (Control): Mice continued to be fed a standard laboratory diet (SLD) after acclimatization. After 7 weeks of feeding, the mice were fasted (with free access to water) for 12 h and intraperitoneally injected with sodium citrate buffer at 8:00 a.m. for 4 consecutive days.

MD group (T2DM with MASLD mice) (26, 27): After acclimatization, mice were switched to a high-fat diet (HFD, 33.35% fat, 27.91% carbohydrate, 23.93% protein by weight, total energy 4.98 kcal/g). After 7 weeks of HFD, the mice were fasted (with free access to water) for 12 h and intraperitoneally injected with a low dose of Streptozocin (STZ) at 40 mg/kg at 8:00 a.m. for 4 consecutive days. One week later, random blood glucose levels were measured from the tail vein. All 6 mice exhibited blood glucose levels higher than 11.1 mmol/L, confirming successful induction of the T2DM model.

MF group (MASLD-only model): After acclimatization, mice were switched to a HFD. After 7 weeks of HFD, the mice were fasted (with free access to water) for 12 h and intraperitoneally injected with sodium citrate buffer at 8:00 a.m. for 4 consecutive days.

2 Validation experiment

After 1 week of acclimatization, 20 mice were randomly divided into 2 groups (n = 10 per group) as follows:

*Normal mice*: After 1 week of acclimatization, mice continued to be fed a SLD. After 7 weeks of feeding, the mice were fasted (with free access to water) for 12 h and intraperitoneally injected with sodium citrate buffer at 8:00 a.m. for 4 consecutive days. Before the end of the experiment, 5 randomly selected normal mice were intraperitoneally injected with saline at 8:00 a.m. for 4 consecutive days and designated as the NS group (N + Saline treated). The remaining 5 normal mice were intraperitoneally injected with 100 mg/kg *α*-HB (TargetMol, Shanghai, China) at the same time and designated as the NHB group (N + α-HB treated).

*T2DM with MASLD mice*: After 1 week of acclimatization, mice were switched to a HFD. After 7 weeks of high-fat feeding, the mice were fasted (with free access to water) for 12 h and intraperitoneally injected with a low dose of STZ at 40 mg/kg at 8:00 a.m. for 4 consecutive days. One week later, random blood glucose levels were measured from the tail vein. All mice exhibited blood glucose levels higher than 11.1 mmol/L, confirming successful induction of the T2DM model. Before the end of the experiment, 5 randomly selected T2DM with MASLD mice were intraperitoneally injected with normal saline at 8:00 a.m. for 4 consecutive days and designated as the DS group (D + Saline treated). The remaining 5 T2DM with MASLD mice were intraperitoneally injected with *α*-HB at 100 mg/kg at the same time and designated as the DHB group (D + α-HB treated).

3 Experimental endpoint and data recording

All experimental mice were maintained until week 12, mice in the DS and DHB groups were kept on a HFD. Throughout this period, the overall health status, diurnal activity rhythms, and feeding behavior of the animals were closely monitored. Meanwhile, body weight changes and random blood glucose levels of mice in each group were measured and meticulously recorded at fixed time points each week.

### 2.3 Specimen collection and storage

The mice were fasted for 8–12 h and anesthetized by intraperitoneal injection of 0.5% pentobarbital sodium solution. After anesthesia, blood and liver tissues were collected. Blood samples from C57 mice in the CON, MD, and MF groups were subjected to metabolomic analysis, while liver samples were used for transcriptomic analysis and oil red O staining. Blood samples from mice in the NS, NHB, DS, and DHB groups were used for biochemical analysis, and liver samples were used for experiments including Western blot (WB), quantitative real-time PCR (QPCR), oil red O staining, hematoxylin and eosin (HE) staining, and immunohistochemistry. Liver specimens for pathological staining were fixed with paraformaldehyde, and all other samples were stored at −80 °C and thawed on ice prior to use.

### 2.4 Metabolomic analysis

For metabolomic analysis, 100 μL of murine serum was aliquoted into methanol solution (0.3 mL) containing 5 μL internal standard (ribitol, 5 mg/mL), vortex-mixed for 1 min, sonicated at 4 °C for 30 min, and centrifuged at 12,000 rpm for 10 min. Subsequently, 200 μL supernatant was transferred to 1.5-mL microtubes, concentrated by rotary evaporation, lyophilized, and derivatized with 35 μL methoxyamine hydrochloride in pyridine (20 mg/mL) under 30-s vigorous vortexing prior to 90-min incubation at 37 °C. Following addition of 35 μL BSTFA (with 1% TMCS), samples were reacted at 70 °C for 60 min and equilibrated at ambient temperature for 30 min before GC–MS analysis. Chromatographic separation was performed on a Thermo Scientific ISQ 7000/Trace 1300_1310 system equipped with an Agilent J&W Scientific DB-5 chromatographic column (60 m × 0.25 mm × 0.25 μm). Instrumental parameters were configured as follows: injector temperature 280 °C, electron ionization (EI) ion source temperature 230 °C, ultra-high purity helium (≥99.999%) carrier gas at constant flow, split ratio 5:1, injection volume 1.0 μL, and solvent delay 5 min. The temperature gradient program initiated at 70 °C, ramped at 10 °C min^−1^ to 200 °C, followed by a 5 °C min^−1^ ascent to 280 °C with a 10-min final hold. Mass spectral acquisition employed full scan mode across 30–550 (m/z). Post-acquisition data processing in Chromeleon 7.0 (Thermo Scientific) encompassed peak feature extraction, spectral annotation against the NIST 2020 Mass Spectral Library (Version 17), and subsequent curation in Microsoft Excel 2016 to assemble a two-dimensional data matrix containing Retention times, observational variables (samples), and corresponding peak intensities. Significantly differential metabolites were defined based on the following criteria: Fold change (FC) threshold: |log₂FC| ≥ 0; Statistical significance: *p*-value < 0.05 from univariate tests (Student’s *t*-test); Multiple testing correction: Adjusted p-value (FDR) < 0.05 using the Benjamini-Hochberg method. Metabolites simultaneously satisfying these criteria were classified as significantly altered.

### 2.5 RNA sequencing

Total RNA was isolated utilizing the Trizol reagent kit (Invitrogen, Carlsbad, CA, USA) in accordance with the instructions provided by the manufacturer. The integrity of the RNA was evaluated using an Agilent 2100 Bioanalyzer (Agilent Technologies, Palo Alto, CA, USA) and further confirmed through RNase-free agarose gel electrophoresis. Following the extraction of total RNA, eukaryotic mRNA was selectively enriched using Oligo(dT) beads. Subsequently, the enriched mRNA was fragmented into shorter segments with the aid of a fragmentation buffer and then reverse transcribed into complementary DNA (cDNA) employing the NEB-Next Ultra RNA Library Prep Kit for Il-lumina (NEB #7530, New England Biolabs, Ipswich, MA, USA). The purified double-stranded cDNA fragments underwent end repair, the addition of an A base, and ligation to Illumina sequencing adapters. The ligation products were purified using AMPure XP Beads (1.0X). The ligated fragments were then subjected to size selection via agarose gel electrophoresis and amplified through polymerase chain reaction (PCR). The resulting cDNA library was sequenced on the Illumina Novaseq 6000 platform by Gene Denovo Biotechnology Co. (Guangzhou, China). Significantly differential gene were defined based on the following criteria: Fold change (FC) threshold: |log₂FC| ≥ 1; Statistical significance: *p*-value < 0.05 from univariate tests (Student’s *t*-test); Multiple testing correction: Adjusted p-value (FDR) < 0.05 using the Benjamini-Hochberg method. Genes simultaneously satisfying these criteria were classified as significantly altered.

### 2.6 Biochemical analysis

Serum metabolic and inflammatory markers—including total cholesterol (TC), triglycerides (TG), low-density lipoprotein cholesterol (LDL-C), high-density lipoprotein cholesterol (HDL-C), TNF-*α*, and IL-6—were quantified using commercial ELISA kits per manufacturer protocols (Nanjing Jiancheng Bioengineering Institute, China). Fasting serum insulin was measured with chemiluminescence immunoassays (Beyotime Biotechnology, China).

### 2.7 Histological analysis

Liver tissues were fixed in 4% paraformaldehyde, paraffin-embedded, and sectioned at 5 μm thickness for H&E histology. For lipid quantification, cryosectioned liver tissues were stained with Oil Red O to visualize lipid droplets. Histomorphological assessments across experimental groups were performed using brightfield microscopy.

For GYS2 immunohistochemistry (IHC), sections underwent peroxidase quenching with 3% H₂O₂/methanol (15 min). Following antigen retrieval, tissues were incubated with anti-GYS2 primary antibody (24 h, 4 °C). After PBS washes, sections were probed with HRP-conjugated secondary antibody (1 h, RT). Signal development used DAB chromogen with hematoxylin counterstaining, and images were captured using a motorized light microscope.

### 2.8 Western blot analysis

Protein lysates were isolated from liver tissues using RIPA lysis buffer (Proteintech, Wuhan, China) supplemented with 1% protease and phosphatase inhibitor cocktails (Proteintech, Wuhan, China). Total protein concentration was quantified using a BCA assay kit (Biosharp, Beijing, China). Samples were normalized to equal protein concentrations, mixed with loading buffer, and denatured at 100 °C for 10 min. A total of 20 μg protein per lane was resolved on 10% SDS-PAGE gels and electrophoretically trans-ferred onto nitrocellulose membranes.

Membranes were blocked with 5% non-fat milk or 5% BSA for 90 min at room temperature, followed by incubation with primary antibodies against GYS2 (No. 22371-1-AP, Proteintech, Wuhan, China), GSK3β (No. 22104-1-AP, Proteintech, Wuhan, China), and P-GSK3β (Ser9) (No. 67558-1-Ig, Proteintech, Wuhan, China) overnight at 4 °C. After five 5-min washes with TBST, membranes were probed with horseradish peroxidase (HRP)-conjugated secondary antibodies (No. RGAR001, No. RGAM001, Proteintech, Wuhan, China) for 1 h at room temperature. Protein bands were visualized using an ultrasensitive ECL substrate (Proteintech, Wuhan, China).

### 2.9 Quantitative real-time PCR

Total RNA was extracted from mouse liver using Trizol reagent (Thermo Fisher Scientific, 15596026CN). Subsequently, it was reverse transcribed into cDNA using the Re-verse Transcription Kit (Vazyme, R222-01). For real-time PCR, specific primers were added to the cDNA along with SYBR Green Pre-mix (Vazyme, Q111-02). Amplification was performed using the LightCycler® 480 instrument, and data analysis was conducted accordingly. The primers used for RT-qPCR detection were as follows:

βactin left primer: 5′-TACTGCTCTGGCTCCTAGCA-3′.

βactin right primer: 5′-CGGACTCATCGTACTCCTGC-3′.

GSK3β left primer: 5′-CGGAAGCCGCTGAAAACAAA-3′.

GSK3β right primer: 5′-ACGGAGCAACGGACTTGAAT-3′.

GYS2 left primer: 5′-ACCTCAGATTGCTGGCTCAC-3′.

GYS2 right primer: 5′-CCAAGGGATGTCACCGACAA-3′.

### 2.10 Statistical analysis

Statistical analyses were conducted in GraphPad Prism 6. Data are expressed as mean ± SEM. Comparisons across four experimental groups were performed using either two-way ANOVA (as appropriate to experimental design) with Tukey’s *post hoc* correction for multiple comparisons. Statistical significance was defined as corrected *p* < 0.05.

## 3 Results

### 3.1 Establishment of the model of T2DM with MASLD

**Figure 1:**
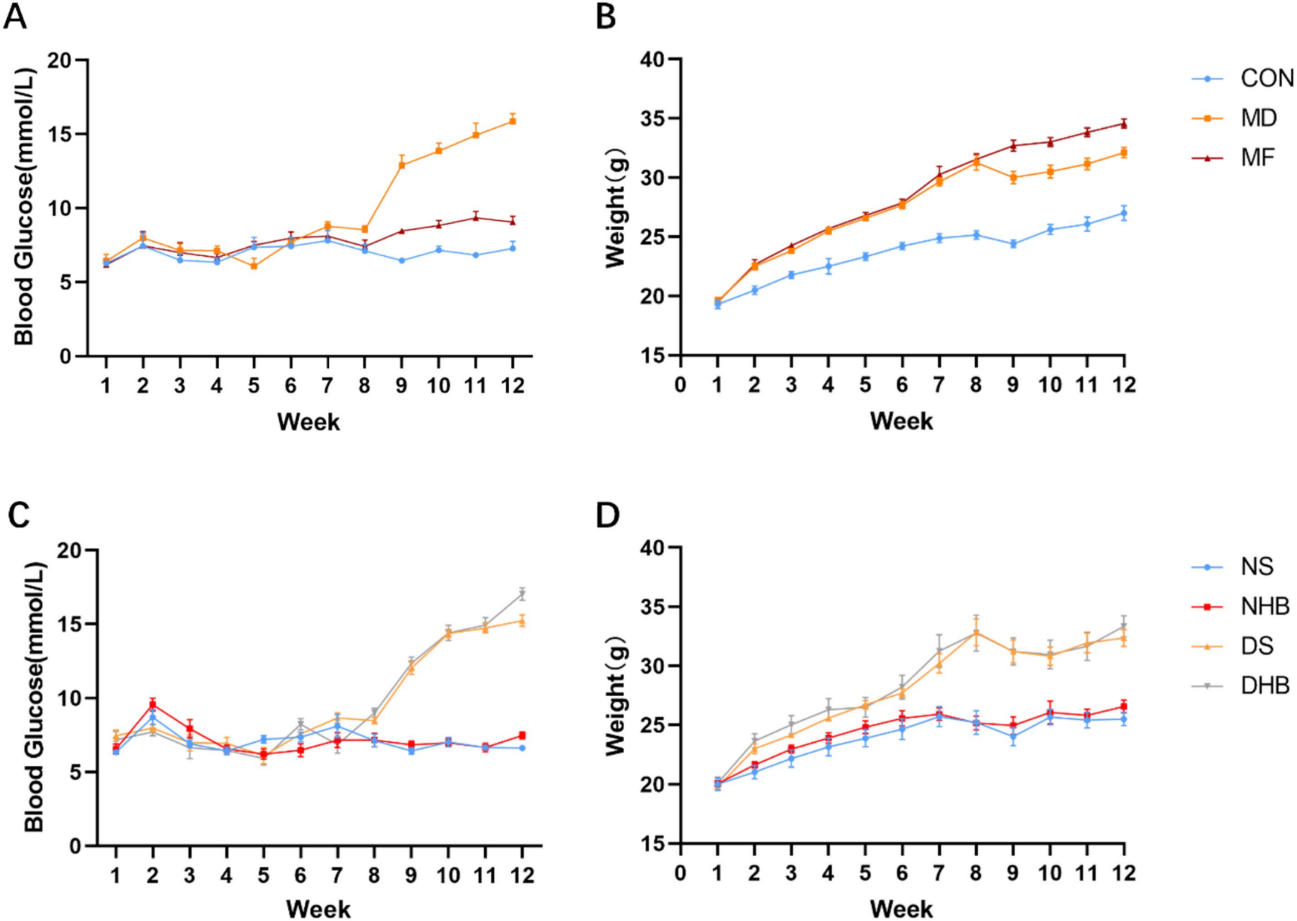
Establishment of the T2DM model. **(A)** Line graph showing the weekly changes in random blood glucose levels in mice from the CON, MD, and MF groups. **(B)** Line graph showing the weekly changes in body weight in mice from the CON, MD, and MF groups. **(C)** Line graph showing the weekly changes in random blood glucose levels in mice from the NS, NHB, DS, and DHB groups. **(D)** Line graph showing the weekly changes in body weight in mice from the NS, NHB, DS, and DHB groups.

As shown in Figure 1, random blood glucose and body weight were monitored weekly. After low-dose STZ treatment, mice in the MD, DS, and DHB groups exhibited significantly elevated random blood glucose levels, indicating the establishment of a stable hyperglycemic phenotype. In contrast, mice in the MF group, which received only a high-fat diet, showed only a mild increase in blood glucose.

**Figure 2:**
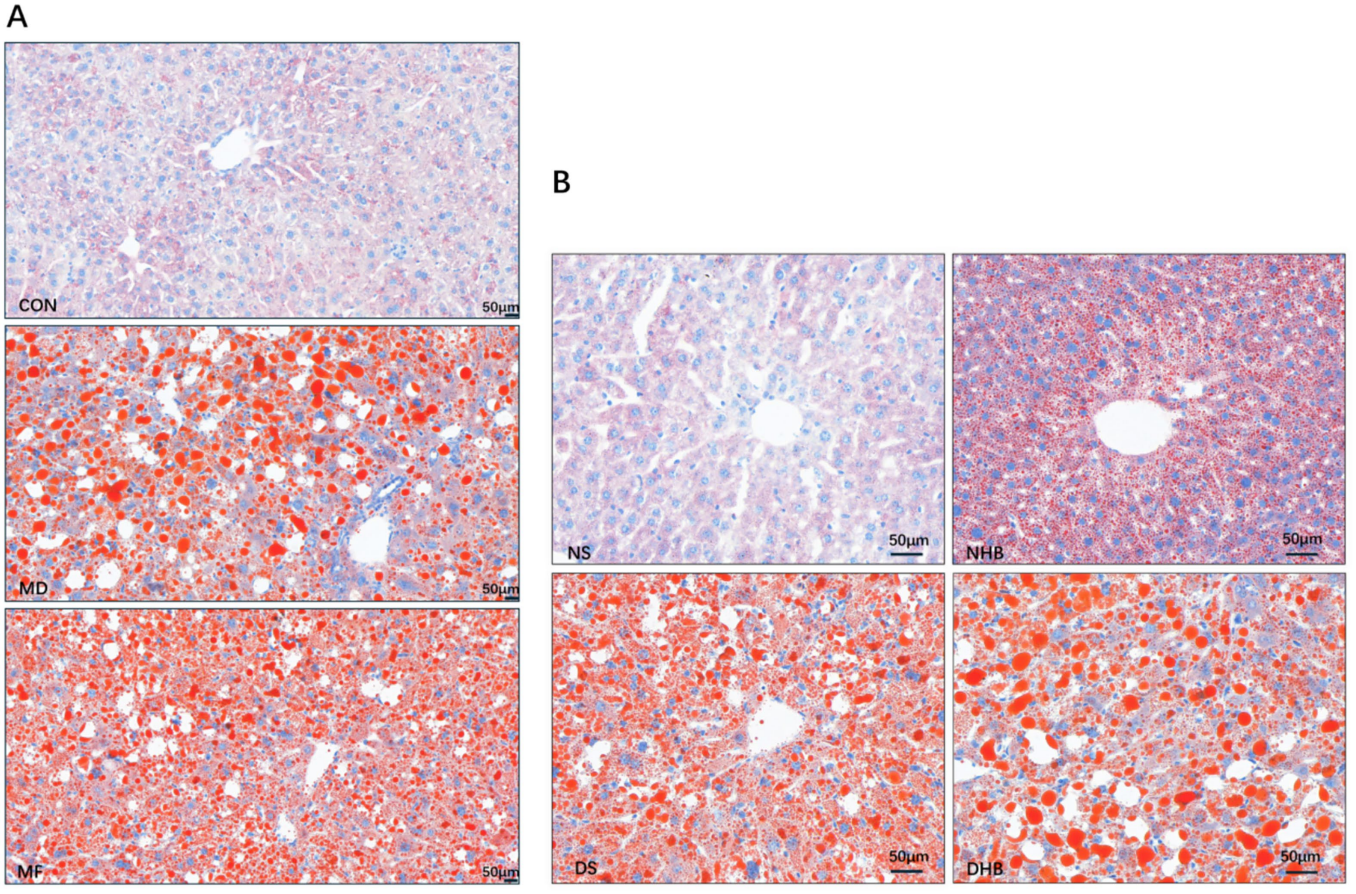
Establishment of the MASLD model. **(A)** Representative images of liver oil red O staining in mice from the CON, MD, and MF groups. **(B)** Representative images of liver oil red O staining in mice from the NS, NHB, DS, and DHB groups.

To further confirm the successful establishment of the MASLD model, liver tissue sections from the mice were subjected to Oil Red O staining. As shown in Figure 2, liver sections from the CON and NS groups displayed normal morphological structure, with hepatocytes arranged in cord-like patterns around the central veins. In contrast, livers from mice in the MD, MF, DS, and DHB groups, which underwent long-term high-fat feeding, exhibited significant lipid deposition. The sections revealed disorganized hepatocellular architecture accompanied by large lipid droplets and vacuoles, indicating successful induction of the MASLD model.

These results demonstrate that mice in the MD, DS, and DHB groups developed severe fatty liver disease on top of a pronounced diabetic phenotype, consistent with the characteristics of a T2DM with MASLD model. The MF group primarily exhibited a simple fatty liver phenotype without T2DM.

### 3.2 Increased serum levels of *α*-HB in the T2DM with MASLD mice

**Figure 3:**
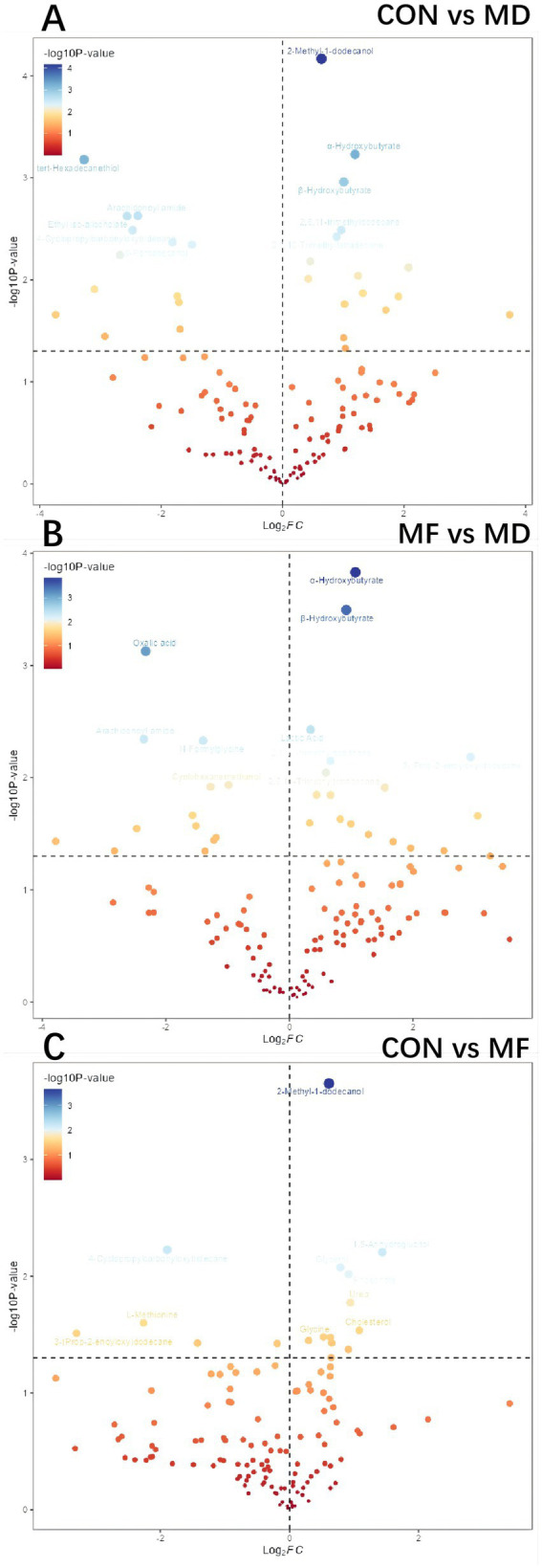
Differential metabolites in mice from the CON, MD, and MF groups. **(A)** Volcano plot showing 16 upregulated and 13 downregulated differential metabolites between the MD and CON groups. **(B)** Volcano plot showing 17 upregulated and 13 downregulated differential metabolites between the MD and MF groups. **(C)** Volcano plot showing 11 upregulated and 5 downregulated differential metabolites between the MF and CON groups.

Following successful validation of the animal models, serum samples were collected from mice in the MD, MF, and CON groups for metabolomic profiling using gas chromatography–mass spectrometry (GC–MS). By applying a significance threshold, metabolites with a *p*-value less than 0.05 were identified as significantly differential metabolites and visualized via volcano plots. Compared to the CON group, 16 metabolites were significantly upregulated and 13 were significantly downregulated in the MD group (Figure 3A). In comparison to the MF group, the MD group exhibited 17 significantly upregulated and 13 significantly downregulated metabolites (Figure 3B). Furthermore, when comparing the MF group to the CON group, 11 metabolites were found to be significantly increased and 5 were significantly decreased (Figure 3C).

Further analysis of the differential metabolite revealed a significant elevation in the serum levels of a ketone body, *α*-HB, in MD group mice. Specifically, the α-HB level in the MD group was increased by 2.3-fold compared to the CON group (*p* < 0.0001) and by 2.1-fold compared to the MF group (*p* < 0.0001). Notably, although no significant difference in α-HB was observed between the MF and CON groups (*p* = 0.4100), the serum α-HB content in the MF group still showed an increasing trend.

**Figure 4:**
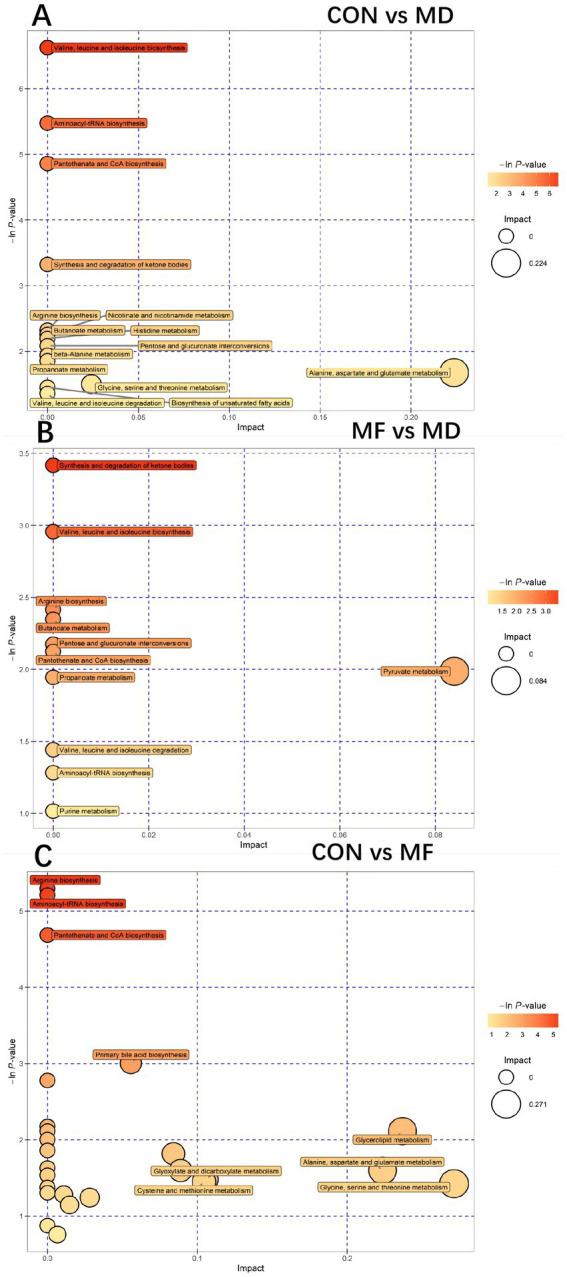
KEGG enrichment analysis of differential metabolites in mice from the CON, MD, and MF groups. In the bubble plot, each bubble represents a metabolic pathway. The horizontal coordinate and the size of each bubble indicate the impact factor of the corresponding pathway in topological analysis, with larger bubbles representing higher impact factors; the vertical coordinate and the color of each bubble represent the *p*-value from enrichment analysis, with deeper colors indicating smaller *p*-values and more significant enrichment. **(A)** Bubble chart showing the pathways enriched by differential metabolites between the MD and CON groups, which are primarily concentrated in glucose and lipid metabolism pathways. **(B)** Bubble chart showing the pathways enriched by differential metabolites between the MD and MF groups, which are primarily concentrated in ketone body metabolism. **(C)** Bubble chart showing the pathways enriched by differential metabolites between the MF and CON groups, which are primarily concentrated in lipid metabolism pathways.

To elucidate the biological implications of these differential metabolites, KEGG metabolic pathway analysis was performed to identify key pathways closely associated with the observed metabolite alterations. The results indicated that the differential metabolites between the MD and CON groups were primarily involved in metabolic processes, including amino acid metabolism, glucose metabolism, and lipid metabolism (Figure 4A). Compared to the MF group, the MD group exhibited more severe disruption in the ketone body metabolism pathway (Figure 4B). Additionally, the differential metabolites in the MF group were predominantly enriched in the triglyceride metabolism pathway, consistent with the pathological feature of hepatic lipid accumulation in this model (Figure 4C). Compared to the CON and MF groups, mice in the MD group demonstrated more extensive metabolite alterations and more severe metabolic pathway disturbances.

In summary, metabolomic profiling revealed a marked elevation in serum *α*-HB levels in T2DM with MASLD mice, accompanied by widespread disturbances in amino acid, glucose, and lipid metabolism pathways.

### 3.3 Downregulation of GYS2 expression is associated with elevated α-HB in the T2DM with MASLD mice

**Figure 5:**
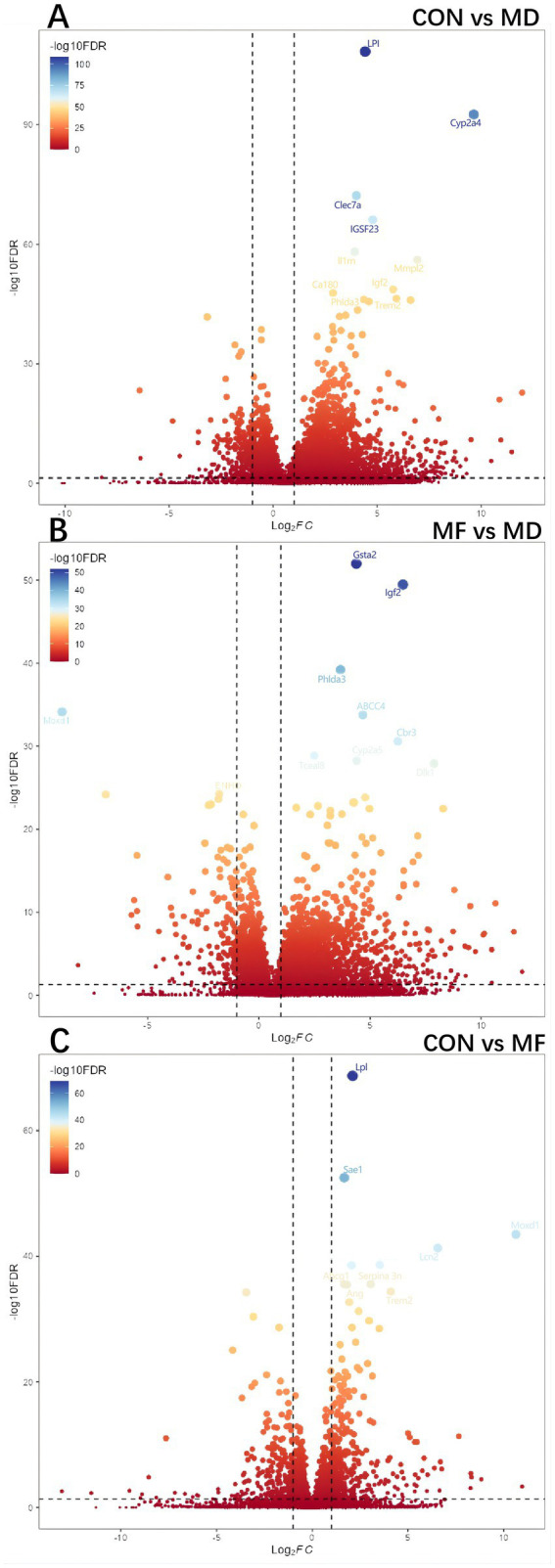
Differential genes and enriched pathways in mice from the CON, MD, and MF groups. **(A)** Volcano plot showing 3,590 upregulated and 260 downregulated differential genes between the MD and CON groups. **(B)** Volcano plot showing 2,934 upregulated and 172 downregulated differential genes between the MD and MF groups. **(C)** Volcano plot showing 475 upregulated and 314 downregulated differential genes between the MF and CON groups.

We further performed high-throughput transcriptome sequencing analysis on liver tissues from mice in the MD, MF, and CON groups. Similar to the metabolomics analysis, we applied stringent thresholds to identify significantly differentially expressed genes (DEGs) between groups, defined as those with |log₂FC| ≥ 1 and an adjusted *p*-value < 0.05, and visualized them using volcano plots. The results revealed extensive transcriptomic remodeling in the MD group compared to the CON group, with 3,590 genes significantly upregulated and 260 genes significantly downregulated (Figure 5A). Compared to the MF group, the MD group also exhibited significant alterations, with 2,934 genes upregulated and 172 genes downregulated (Figure 5B). In contrast, transcriptomic changes between the MF and CON groups were relatively limited, with only 475 genes upregulated and 314 genes downregulated (Figure 5C).

Among the numerous DEGs, a key finding was that GYS2 exhibited a significantly altered expression pattern, showing significant downregulation in both the MD and MF groups. Further comparison between the MD and MF groups indicated that the downregulation of GYS2 was more pronounced in the MD group. It is noteworthy that GYS2 plays a crucial role in hepatic glucose and lipid metabolism, and its downregulation can lead to impaired glycogen synthesis and promote ectopic lipid accumulation.

**Figure 6:**
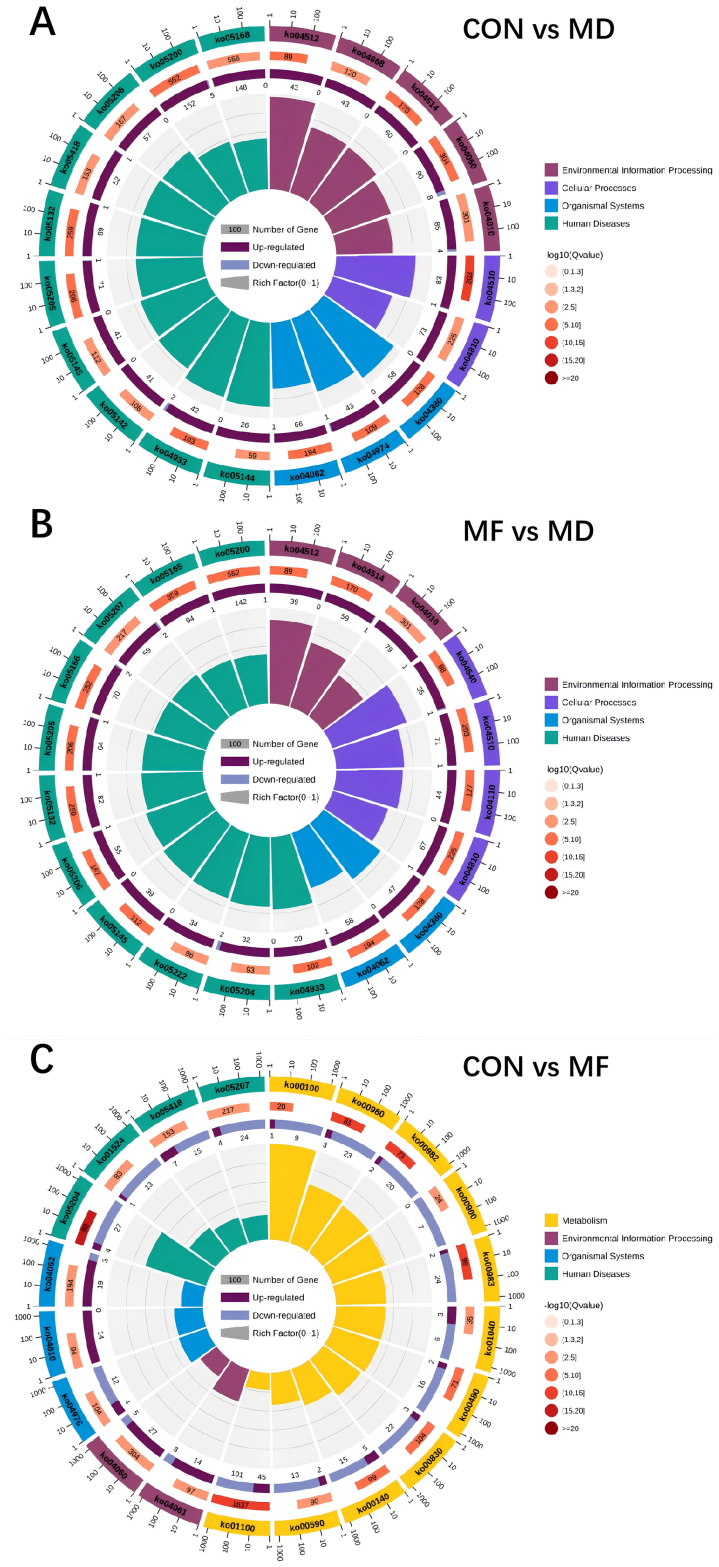
Differential genes enriched pathways in mice from the CON, MD, and MF groups. **(A)** Enrichment circle plot showing the pathways enriched by differential genes between the MD and CON groups. **(B)** Enrichment circle plot showing the pathways enriched by differential genes between the MD and MF groups. **(C)** Enrichment circle plot showing the pathways enriched by differential genes between the MF and CON groups.

Subsequently, KEGG pathway enrichment analysis was performed on the identified DEGs to investigate their potential roles in disease pathogenesis from a functional perspective. The enrichment results showed that upregulated DEGs in the MD group were primarily enriched in pathways related to Human Diseases (e.g., endocrine and metabolic diseases, cardiovascular complications) and Environmental Information Processing (e.g., cytokine-cytokine receptor interaction, various signaling transduction pathways) (Figures 6A,B).

In contrast, the downregulated DEGs in the MF group were mainly enriched in metabolic pathways (Figure 6C), particularly lipid metabolism and amino acid metabolism. Of particular note, comparative analysis revealed that the enrichment of DEGs in inflammatory cytokine signaling pathways was significantly higher in the MD group than in the MF group.

**Figure 7:**
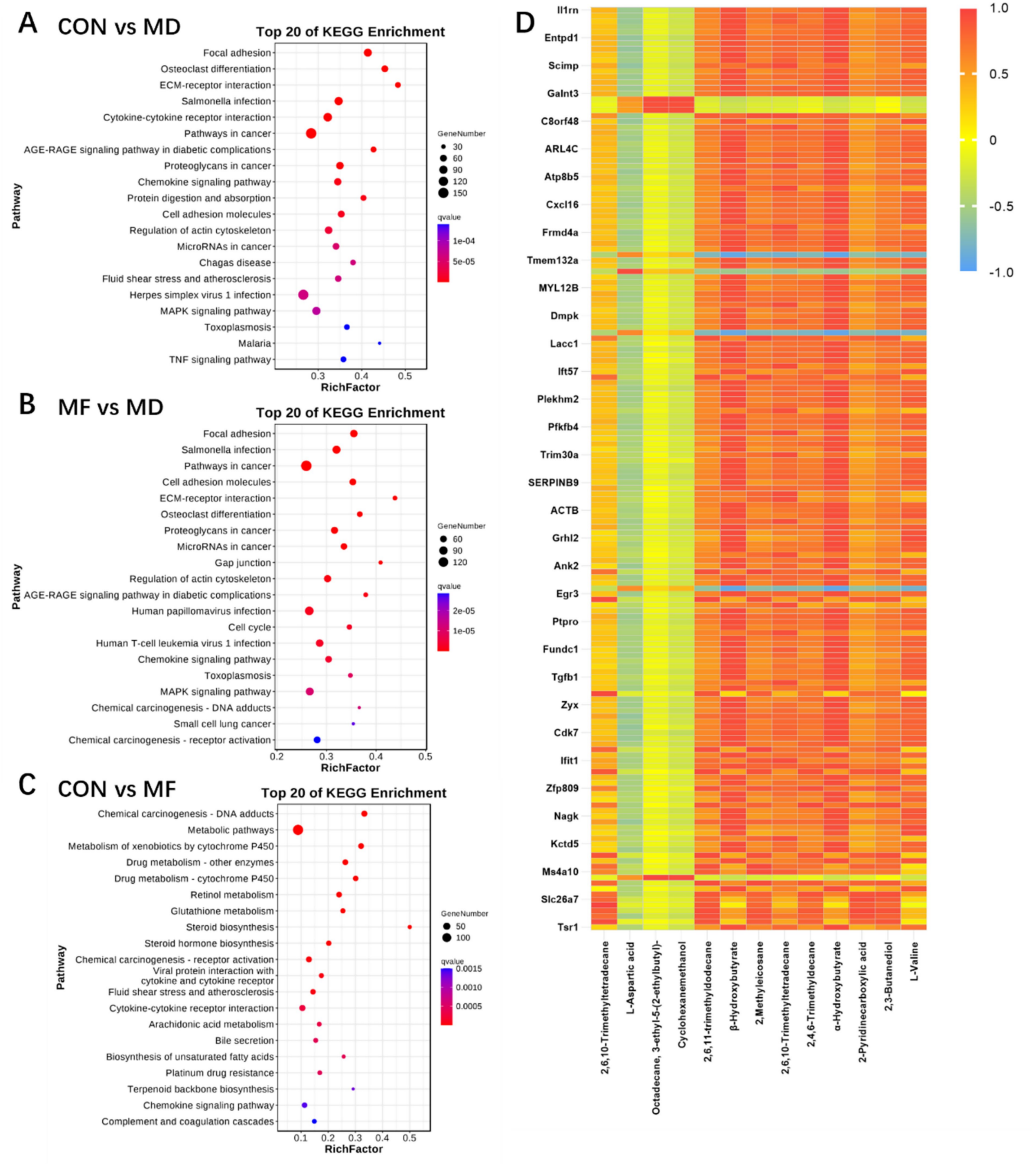
Correlation analysis between metabolomics and transcriptomics. **(A)** Bubble chart showing the top 20 pathways enriched by differential genes between the MD and CON groups. **(B)** Bubble chart showing the top 20 pathways enriched by differential genes between the MD and MF groups. **(C)** Bubble chart showing the top 20 pathways enriched by differential genes between the MF and CON groups. **(D)** Heatmap showing significantly correlated differential metabolites and genes from metabolomics and transcriptomics.

To further delineate the functional enrichment of DEGs across groups, we selected the top 20 KEGG enrichment pathways with the smallest *p*-values and visualized them using bubble plots. Interpretation of the results revealed that, compared to the CON and MF groups, DEGs in the MD group mice were significantly enriched in the AGE-RAGE signaling pathway. This pathway is among the most classical pathways implicated in diabetic complications (28, 29). It perpetuates the activation of inflammatory signals, directly leading to oxidative stress, inflammatory responses, and tissue damage, serving as a critical bridge connecting hyperglycemia to hepatic inflammation and fibrosis in fatty liver disease. The remaining genes were primarily concentrated in core pathways involving inflammation and immune regulation, cell stress and death-related pathways, and extracellular matrix remodeling and fibrosis pathways (Figures 7A,B).

In contrast, the differential genes in the MF group were mainly enriched in pathways such as lipid biosynthesis, cytochrome P450 metabolism, and glutathione metabolism (Figure 7C). This pattern reveals a state of lipid metabolic overload and a strongly activated hepatic detoxification system in this model (30, 31).

Finally, to integrate the metabolomic and transcriptomic data, we calculated Pearson correlation coefficients to screen for significantly correlated differential metabolites and genes. Among these, we observed a significant correlation between the increase in *α*-HB and the decrease in GYS2 expression (Figure 7D). Based on these results, we hypothesize that *α*-HB plays an important role in the pathogenesis of T2DM with MASLD and is closely associated with the downregulation of GYS2 expression.

Transcriptomic analysis revealed extensive transcriptional reprogramming in the livers of T2DM with MASLD mice, characterized by marked downregulation of GYS2 and enrichment of differentially expressed genes in pathways related to inflammation, immunity, and diabetic complications. Furthermore, correlation analysis indicated a significant association between elevated *α*-HB levels and downregulated GYS2 expression.

### 3.4 α-HB exacerbates disease progression in T2DM with MASLD mice

**Figure 8:**
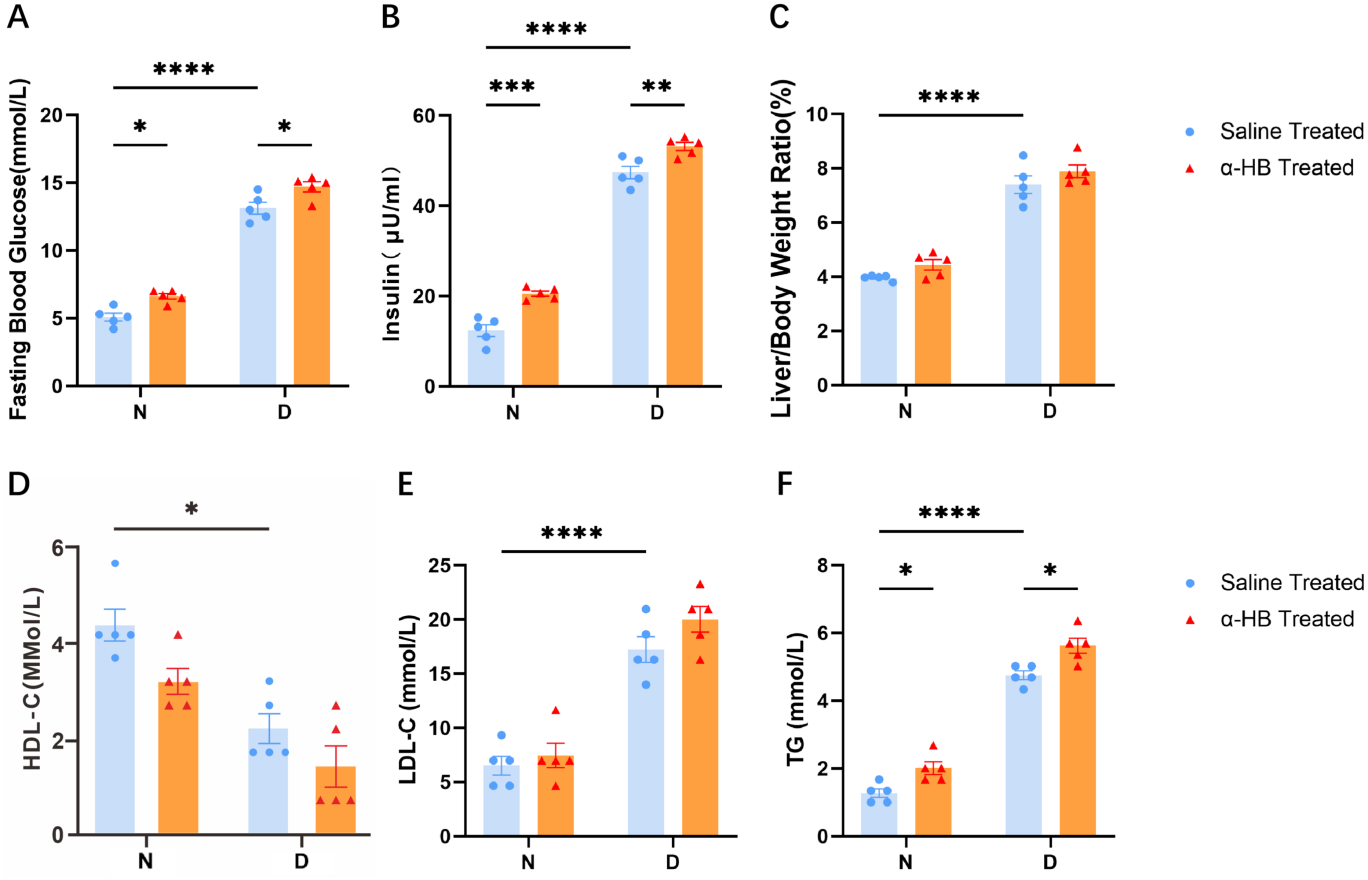
Exacerbation of glucose and lipid metabolism disorders in T2DM with MASLD mice after *α*-HB intervention. In bar graphs, N denotes normal mice, and D denotes T2DM with MASLD mice, both subjected to saline or α-HB intervention. **(A)** Bar graph showing differences in fasting blood glucose levels among groups after α-HB intervention. **(B)** Bar graph showing differences in fasting insulin levels among groups after α-HB intervention. **(C)** Bar graph showing changes in the liver-to-body weight ratio among groups after α-HB intervention. **(D)** Bar graph showing changes in serum high-density lipoprotein (HDL-C) levels among groups after α-HB intervention. **(E)** Bar graph showing changes in serum low-density lipoprotein (LDL-C) levels among groups after α-HB intervention. **(F)** Bar graph showing changes in serum triglyceride (TG) levels among groups after α-HB intervention. Data are presented as mean ± SEM. *p < 0.05, **p < 0.01, ***p < 0.001, ****p < 0.0001 indicate significant differences.

Compared to the NS group, the DS group exhibited a significant increase in fasting blood glucose (FBG) levels (13.1400 ± 0.4411 mmol/L vs. 5.0800 ± 0.2956 mmol/L, *p* < 0.0001), consistent with the hyperglycemic phenotype of the T2DM model. Furthermore, after *α*-HB intervention, the FBG level in the NHB group was 6.6200 ± 0.2035 mmol/L, significantly higher than that in the NS group (*p* = 0.0249). The DHB group also showed a markedly higher FBG level (14.700 ± 0.3674 mmol/L) compared to the DS group (*p* = 0.0230) (Figure 8A). Significant differences in fasting insulin levels were also observed among the four groups (Figure 8B), with the DS group showing significantly higher insulin levels than the NS group (*p* < 0.0001). Additionally, α-HB intervention led to a significant increase in serum insulin levels in both the DHB group (53.1600 ± 0.8946 μU/mL, *p* = 0.0086) and the NHB group (20.5500 ± 0.5852 μU/mL, *p* = 0.0004) compared to their respective control groups. This finding suggests decreased insulin sensitivity in mice following *α*-HB intervention.

Analysis of the calculated liver-to-body weight ratio (Figure 8C) revealed that it was significantly higher in the DS group compared to the NS group (*p* < 0.0001). Although the liver-to-body weight ratio showed a slight increase in the NHB group (*p* = 0.4456) and DHB group (*p* = 0.4338) compared to their controls, the differences did not reach statistical significance.

Measurement of classic lipid profiles showed that the serum high-density lipoprotein cholesterol (HDL-C) level in the NS group was 4.2710 ± 0.3291 mmol/L, significantly higher than that in the DS group (*p* < 0.0001) (Figure 8D), while the low-density lipoprotein cholesterol (LDL-C) level was significantly lower (6.5240 ± 0.8718 mmol/L, *p* < 0.0001) (Figure 8E). Although *α*-HB intervention did not cause significant changes in cholesterol levels, as shown in Figure 8F, the serum triglyceride (TG) level in the DHB group increased to 5.6280 ± 0.5013 mmol/L, significantly higher than the 4.7530 ± 0.1286 mmol/L in the DS group (*p* = 0.0106). The TG level in the NHB group mice (2.0100 ± 0.1835 mmol/L) was also significantly elevated compared to the NS group (1.2730 ± 0.1253 mmol/L, *p* = 0.0334).

**Figure 9:**
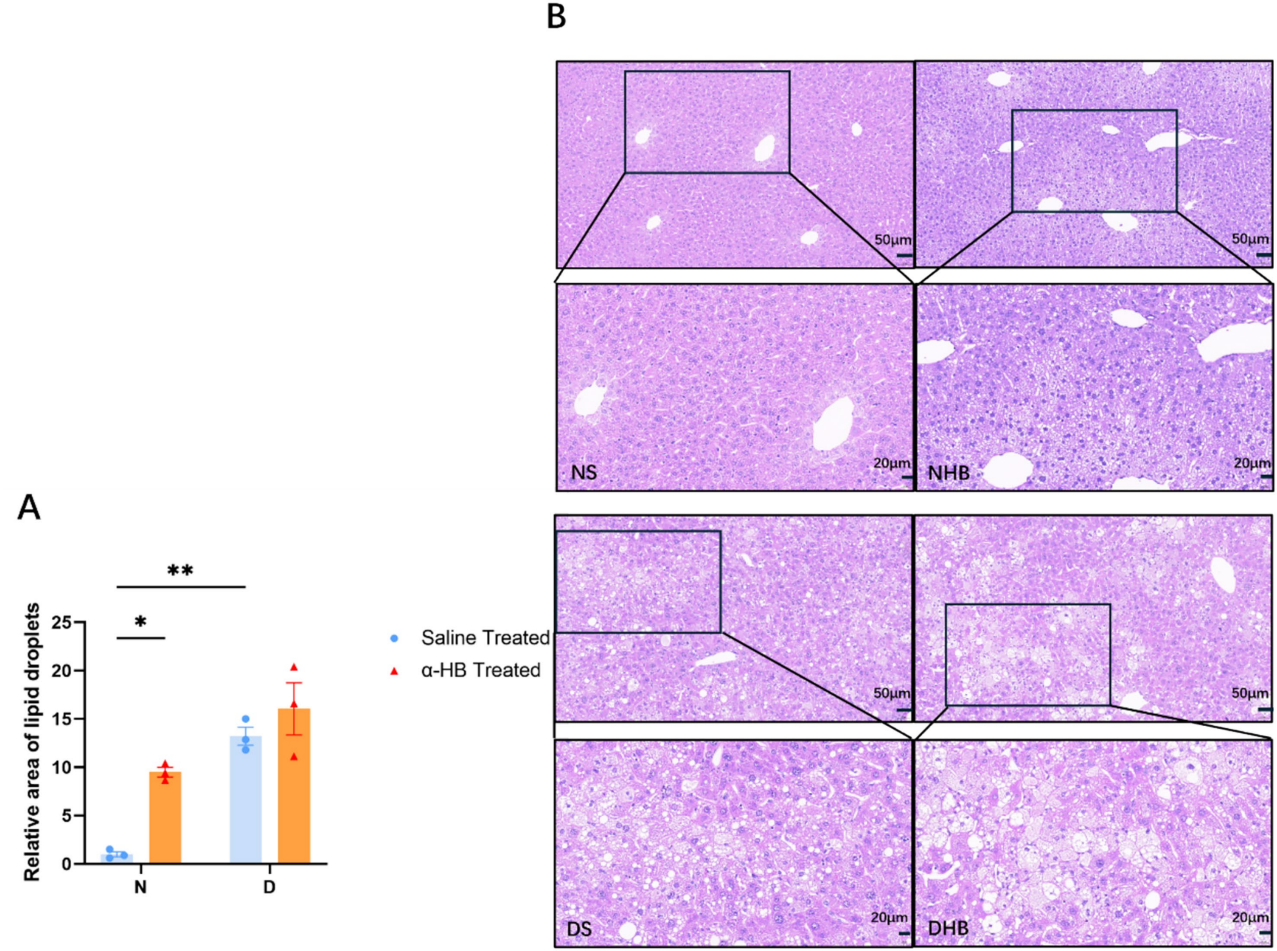
Exacerbation of hepatic steatosis in T2DM with MASLD mice after α-HB intervention. In bar graphs, N denotes normal mice, and D denotes T2DM with MASLD mice, both subjected to saline or α-HB intervention. **(A)** Bar graph showing the difference in the percentage area of lipid droplets after Oil Red O staining among the NS, NHB, DS, and DHB groups after α-HB intervention. **(B)** Representative images of liver H&E staining in mice from the NS, NHB, DS, and DHB groups. Data are presented as mean ± SEM. *p < 0.05, **p < 0.01, ***p < 0.001, ****p < 0.0001 indicate significant differences.

In the earlier validation of successful disease model establishment, Oil Red O staining had already revealed hepatic steatosis in the NHB, DS, and DHB groups. To analyze whether there were significant differences in the severity of hepatic lipid deposition among groups, we performed quantitative analysis on the Oil Red O staining results of liver tissues and estimated the relative area of lipid droplets (Figure 9A). The results showed that the lipid droplet area in liver sections from NS group mice was minimal, only 1/13th of that in the DS group (*p* = 0.0021). After *α*-HB intervention, the lipid droplet area in the NHB group increased markedly compared to the NS group, being 9.5 times larger (*p* = 0.0198). Although the lipid droplet area in the DHB group increased compared to the DS group, the difference did not reach statistical significance (*p* = 0.7517).

Simultaneously, to assess overall pathological changes such as hepatocyte necrosis, inflammation, and fibrosis, HE staining was also performed (Figure 9B). Observation of the stained sections revealed that hepatocytes in the NS group had clear boundaries, with large, round, centrally located nuclei and abundant cytoplasm. In contrast, the NHB group exhibited dense lipid vacuoles. Both the DS and DHB groups showed severe lipid degeneration, with numerous vacuoles within hepatocytes, nuclei and scant cytoplasm displaced to the cell periphery, along with visible inflammatory cell infiltration. The tissue texture appeared foamy due to the abundance of fat vacuoles.

Exogenous *α*-HB administration worsened hyperglycemia, insulin resistance, dyslipidemia, and hepatic steatosis in both T2DM with MASLD and normal mice, indicating its role in aggravating metabolic dysfunction.

### 3.5 *α*-HB exacerbates the levels of inflammatory factors in T2DM with MASLD mice

Previous studies have clearly indicated a definitive association between α-HB and elevated levels of pro-inflammatory cytokines (69). In our study, serum was collected from NS, NHB, DS, and DHB groups, and the levels of pro-inflammatory cytokines were measured, with a particular focus on TNF-α and IL-6.

**Figure 10:**
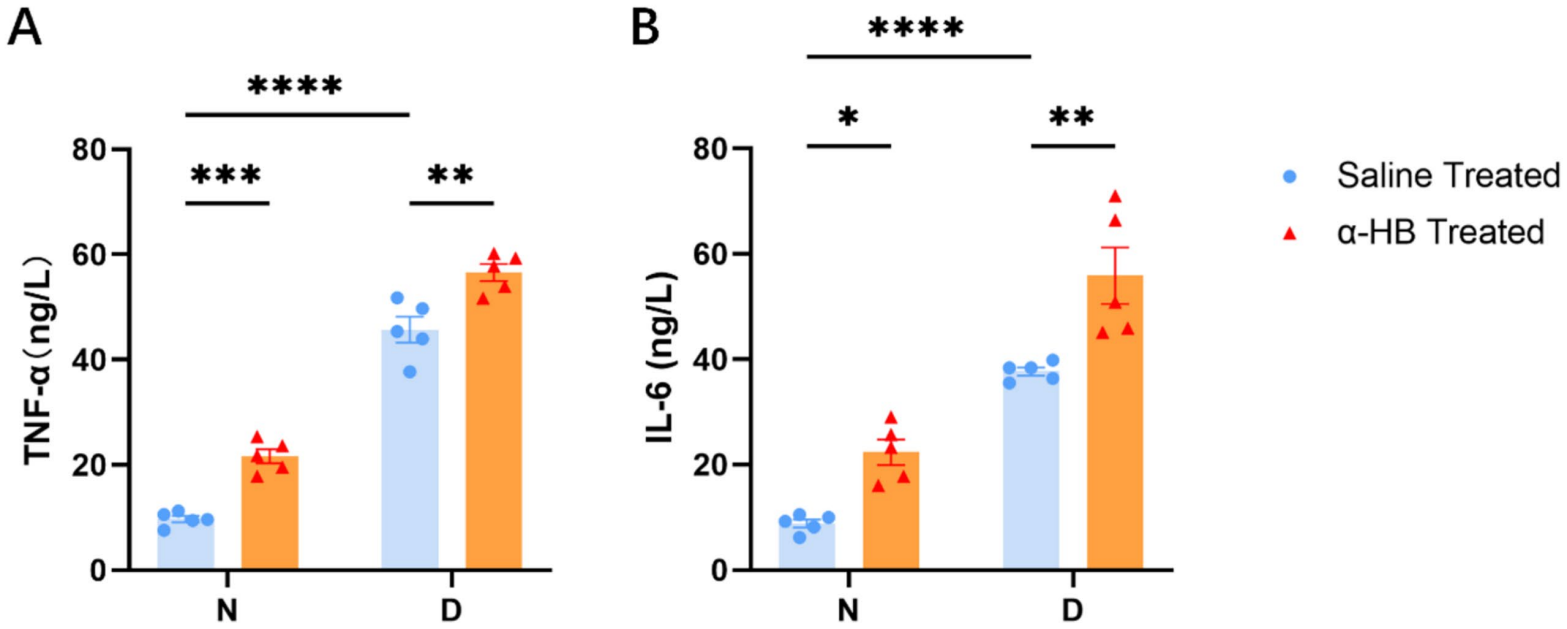
Exacerbation of chronic inflammation in T2DM with MASLD mice after α-HB intervention. In bar graphs, N denotes normal mice, and D denotes T2DM with MASLD mice, both subjected to saline or α-HB intervention. **(A)** Bar graph showing changes in serum tumor necrosis factor-α (TNF-α) levels among groups after α-HB intervention. **(B)** Bar graph showing changes in serum interleukin-6 (IL-6) levels among groups after α-HB intervention. Data are presented as mean ± SEM. *p < 0.05, **p < 0.01, ***p < 0.001, ****p < 0.0001 indicate significant differences.

As shown in Figure 10A, TNF-α levels were significantly higher in both the NHB (21.6300 ± 1.3510 ng/L, *p* = 0.0005) and DS (45.7000 ± 2.4990 ng/L, *p* < 0.0001) groups compared to the NS group. Furthermore, α-HB intervention led to a further increase in TNF-α levels in the DHB group (56.5700 ± 1.6220 ng/L) compared to the DS group (*p* = 0.0013).

Notably, the trend of IL-6 changes in mice was consistent with that of TNF-α. As illustrated in Figure 10B, compared to the NS group (8.8060 ± 0.7702 ng/L), IL-6 levels were also significantly elevated in the NHB (22.3500 ± 2.4190 ng/L, *p* = 0.03) and DS (37.6800 ± 0.7770 ng/L, *p* < 0.0001) groups. Moreover, the IL-6 level in the DHB group (55.8700 ± 5.3940 ng/L) was further increased relative to the DS group (*p* = 0.0029).

α-HB treatment significantly elevated serum levels of pro-inflammatory cytokines TNF-α and IL-6, highlighting its potential to amplify systemic inflammation in T2DM with MASLD.

### 3.6 α-HB downregulates hepatic GYS2 expression and severely disrupts glycogen and lipid metabolism in T2DM with MASLD mice

**Figure 11:**
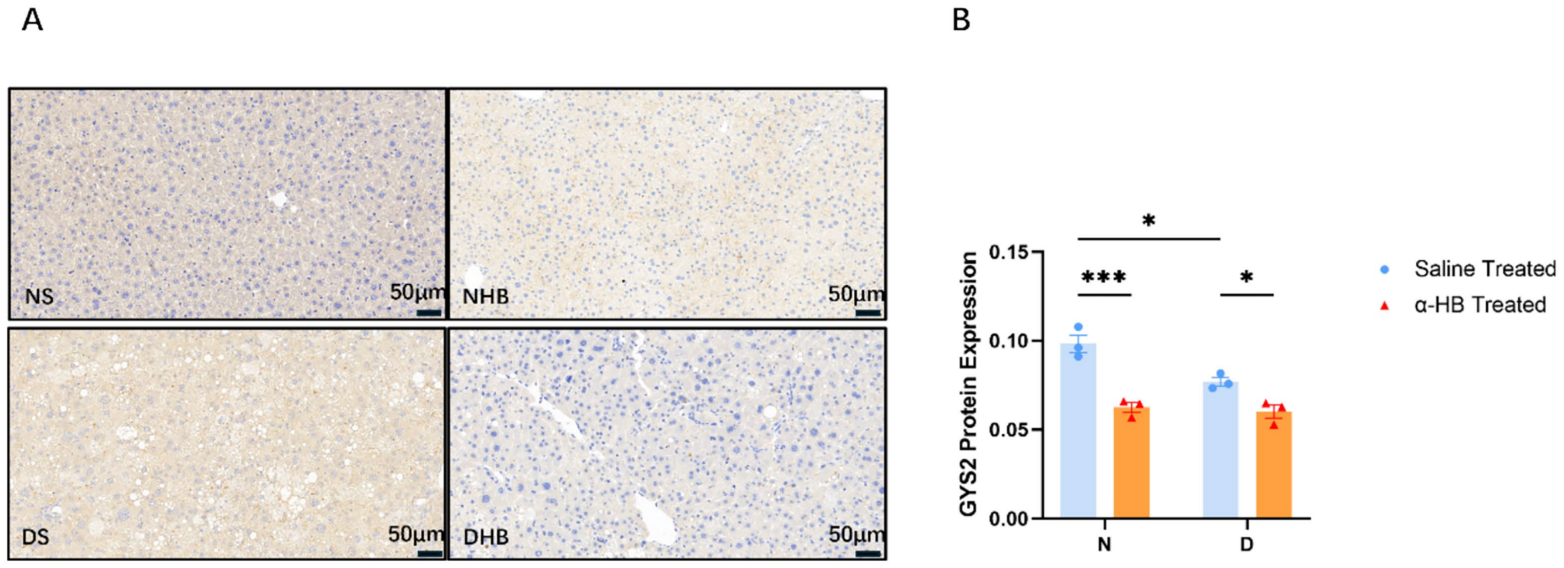
Immunohistochemical analysis of mice from the NS, NHB, DS, and DHB groups. In bar graphs, N denotes normal mice, and D denotes T2DM with MASLD mice, both subjected to saline or α-HB intervention. **(A)** Representative immunohistochemical images of the NS, NHB, DS, and DHB groups. Higher GYS2 expression is indicated by darker brown cytoplasmic staining. **(B)** Bar graph showing semi-quantitative analysis of GYS2 expression differences among groups based on immunohistochemical results. Data are presented as mean ± SEM. *p < 0.05, **p < 0.01, ***p < 0.001, ****p < 0.0001 indicate significant differences.

GYS2, a key enzyme regulating hepatic glycogen synthesis, plays a central role in maintaining glucose homeostasis and is closely associated with various metabolic disorders. To investigate the effect of α-HB intervention on hepatic GYS2 expression, we first performed liver immunohistochemical analysis (Figure 11A). We observed that hepatocyte cytoplasm in liver sections from NS group mice stained deep brown, indicating normal GYS2 expression. In contrast, immunohistochemical staining in the NHB group appeared light brownish-yellow, suggesting decreased GYS2 expression compared to the NS group. Consistently, GYS2 antigen expression in the DHB group was also lower than that in the DS group. To enhance the scientific rigor of the immunohistochemistry results, we further evaluated the staining using a semi-quantitative method (Figure 11B). The scoring results were consistent with the visually observed staining trends. Compared to normal mice in the NS group, GYS2 expression in the livers of DS group mice was significantly downregulated (*p* = 0.0127). Notably, GYS2 levels in the NHB group were significantly lower than those in the NS group (*p* = 0.0005), and GYS2 expression in the DHB group was further reduced compared to the DS group (*p* = 0.0437).

**Figure 12:**
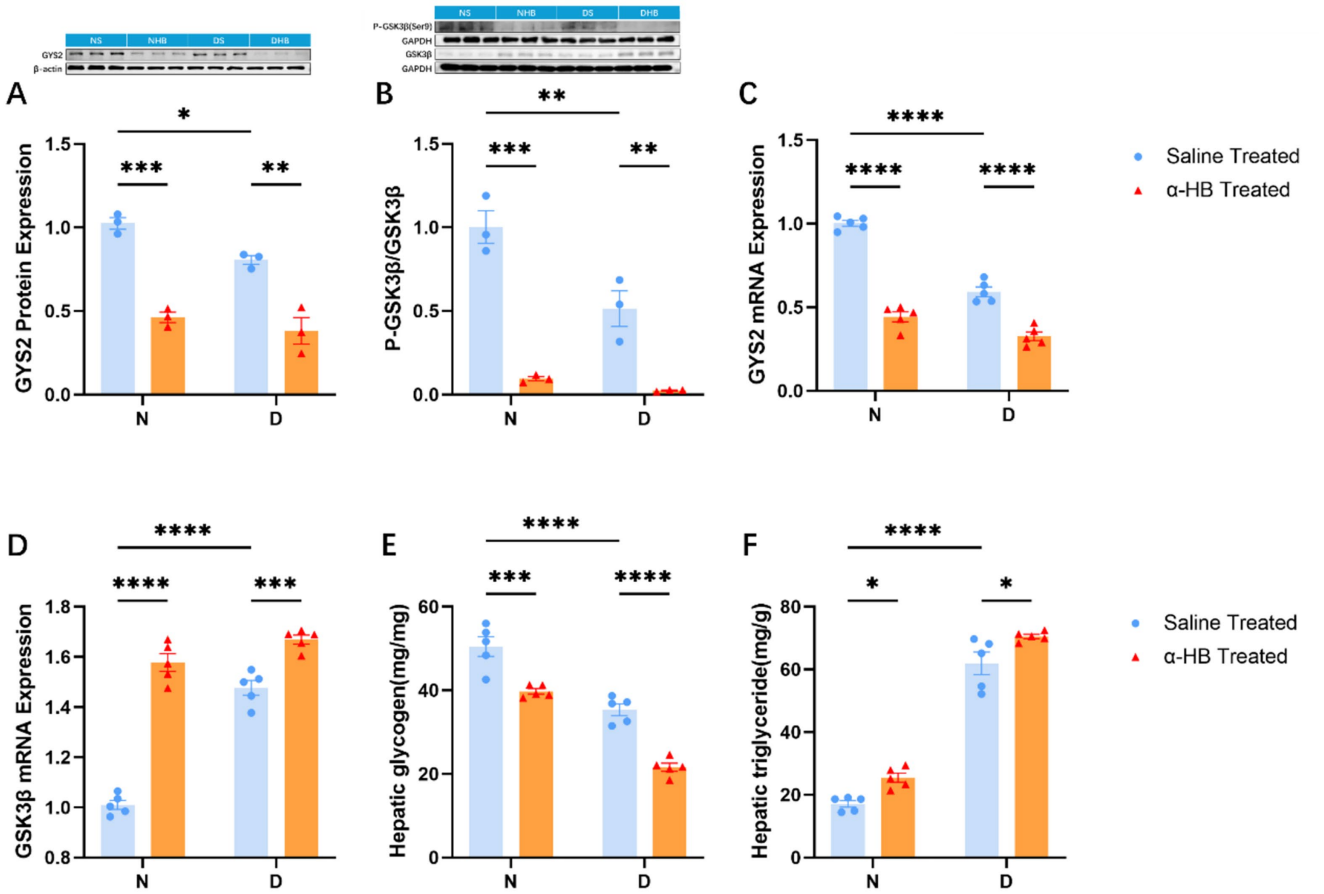
Decreased GYS2 expression accompanied by hepatic glycogen and lipid metabolism disorders in T2DM with MASLD mice after α-HB intervention. In bar graphs, N denotes normal mice, and D denotes T2DM with MASLD mice, both subjected to saline or α-HB intervention. **(A)** Western blot showing hepatic GYS2 protein expression in the NS, NHB, DS, and DHB groups after α-HB intervention. The bar graph shows the differences in hepatic GYS2 expression among these groups. **(B)** Western blot showing hepatic GSK3β and P-GSK3β protein expression in the NS, NHB, DS, and DHB groups after α-HB intervention. The bar graph shows the differences in the P-GSK3β/GSK3β ratio among these groups. **(C)** Bar graph showing differences in hepatic *Gys2* mRNA expression levels among the NS, NHB, DS, and DHB groups. **(D)** Bar graph showing differences in hepatic *Gsk3β* mRNA expression levels among the NS, NHB, DS, and DHB groups. **(E)** Bar graph showing differences in hepatic glycogen content among the NS, NHB, DS, and DHB groups. **(F)** Bar graph showing differences in hepatic triglyceride (TG) content among the NS, NHB, DS, and DHB groups. Data are presented as mean ± SEM. *p < 0.05, **p < 0.01, ***p < 0.001, ****p < 0.0001 indicate significant differences.

To validate the above findings, we conducted Western blot (WB) analysis on liver tissue protein extracts. The results were highly consistent with the immunohistochemistry analysis. GYS2 protein expression in the DS group was significantly reduced compared to the NS group (*p* = 0.0480). On this basis, *α*-HB intervention further downregulated GYS2, with levels in both the NHB (*p* = 0.0002) and DHB (*p* = 0.0011) groups showing a further decrease compared to their respective control groups (Figure 12A).

Glycogen synthase kinase-3β (GSK3β) is an important upstream regulator of GYS2. In its active state, GSK3β inhibits glycogen synthesis by affecting the phosphorylation status of GYS2. We measured hepatic levels of phosphorylated GSK3β at Ser9 (P-GSK3β (Ser9)) and total GSK3β, and calculated the P-GSK3β (Ser9)/GSK3β ratio. Since phosphorylation of GSK3β at Ser9 inactivates it, a higher P-GSK3β (Ser9)/GSK3β ratio indicates lower GSK3β activity. The results showed that this ratio was highest in the NS group (indicating the lowest GSK3β activity). Compared to the NS group, this ratio was significantly decreased in the DS group (*p* = 0.006), suggesting enhanced GSK3β activity. Following *α*-HB intervention, the ratio further decreased in both the NHB group (compared to NS, *p* = 0.0001) and the DHB group (compared to DS, *p* = 0.0064), indicating a further enhancement of GSK3β activity (Figure 12B).

Furthermore, to verify these results at the transcriptional level, we extracted liver RNA for QPCR analysis. Compared to the NS group, GYS2 mRNA expression was significantly reduced in the NHB and DS groups. Moreover, GYS2 mRNA expression in the DHB group was significantly lower than that in the DS group (*p* < 0.0001) (Figure 12C). Correspondingly, GSK3β mRNA expression was significantly higher in the NHB and DHB groups compared to the two groups that did not receive *α*-HB intervention (*p* < 0.0001) (Figure 12D), corroborating the WB findings.

Given the important role of GYS2 in hepatic glucose and lipid metabolism, we directly measured hepatic glycogen and TG levels to assess liver metabolic status. The results showed that glycogen content in the DS group was only 35.3400 ± 1.3980 mg/mg, significantly lower than that in the NS group (50.4900 ± 2.3440 mg/mg, *p* < 0.0001). α-HB treatment further reduced hepatic glycogen levels in both the NHB (39.7400 ± 0.6406 mg/mg, *p* = 0.0005) and DHB (21.6000 ± 0.9723 mg/mg, *p* < 0.0001) groups (Figure 12E). Concurrently, α-HB intervention significantly exacerbated intrahepatic lipid deposition, with hepatic TG levels in the NHB (25.4700 ± 1.4530 mg/g, *p* = 0.0462) and DHB (70.5300 ± 0.6941 mg/g, *p* = 0.0398) groups being significantly elevated compared to their respective non-intervention groups (Figure 12F).

Following α-HB intervention, the expression and activity of GYS2 were reduced, while GSK3β activity was enhanced, leading to decreased hepatic glycogen content and increased triglyceride accumulation, underscoring its central role in disrupting hepatic glucose and lipid homeostasis.

## 4 Discussion

The results of this study reveal that the T2DM with MASLD mice exhibited a synergistic increase in FBG and insulin levels, accompanied by significantly enhanced insulin resistance. This finding is consistent with previous clinical studies: insulin resistance is not only one of the core mechanisms in the development and progression of MASLD (32) but also a key pathological feature that is commonly present and further amplified in the comorbidity of T2DM with MASLD (33, 34). In the context of insulin resistance, the liver’s sensitivity to insulin’s suppression of gluconeogenesis decreases, while peripheral tissue glucose uptake and utilization are impaired, collectively leading to a persistent hyperglycemic state (32). At the same time, the T2DM with MASLD model mice displayed a typical atherogenic lipid profile: significant increases in TG and LDL-C, and a marked decrease in HDL-C. HDL-C is involved in reverse cholesterol transport, helping to return excess peripheral cholesterol to the liver for metabolism and clearance, and is generally considered protective (35). In contrast, elevated LDL-C is closely associated with an increased risk of atherosclerosis and cardiovascular events (36). These lipid metabolism disorders, often driven by chronic overnutrition, form an important metabolic basis for the development and progression of MASLD. Clinical studies have also confirmed that similar dyslipidemia (such as high TG and low HDL-C) and higher BMI are significant predictors of MASLD in individuals with T2DM (37–39).

Furthermore, this study observed the coexistence of metabolic disorders and chronic inflammation in the T2DM with MASLD mice: serum levels of TNF-*α* and IL-6 were significantly elevated, consistent with previous observations of increased pro-inflammatory cytokines in T2DM with MASLD patients (40). Previous evidence indicates that pro-inflammatory factors such as TNF-α and IL-6 are closely related to the degree of metabolic disorders and insulin resistance (40) and act as key mediators linking metabolism and inflammation in disease progression. On one hand, insulin resistance and ectopic lipid deposition can activate innate immune-inflammatory pathways, including cGAS/STING/NF-κB, inducing the release of inflammatory factors such as TNF-α and IL-6 (40, 41). On the other hand, persistently elevated inflammatory factors can further interfere with insulin signaling, exacerbate insulin resistance, and amplify glucose and lipid metabolism abnormalities (33).

In T2DM with MASLD, a self-reinforcing vicious cycle exists, fundamentally rooted in dysregulated nutrient handling: insulin resistance drives glucose and lipid metabolism disorders, which induce chronic inflammation. This inflammation, in turn, exacerbates metabolic imbalance and insulin resistance. This metabolism–inflammation positive feedback loop, often initiated and sustained by poor dietary patterns, may be one of the important driving mechanisms for the continuous progression of T2DM with MASLD. Therefore, identifying key metabolites and molecular nodes that connect nutritional stress with gene expression remodeling is of great significance for elucidating the mechanisms underlying the progression of this comorbidity.

Analysis of transcriptomic from normal control mice, T2DM with MASLD mice, and MASLD-only mice in this study revealed that the differentially expressed genes between T2DM with MASLD mice and MASLD mice were significantly enriched in multiple human disease-related pathways. Furthermore, compared to MASLD mice, T2DM with MASLD mice exhibited more pronounced changes in the expression levels of related pathogenic/disease-associated genes.

This result suggests that the metabolic stress environment characterized by “hyperglycemia–hyperlipidemia” formed by superimposing glucose metabolism abnormalities on a high-fat background, a state mirroring prolonged postprandial nutrient excess, may induce more complex and extensive molecular responses and pathological processes. Previous studies have shown that chronic hyperglycemia can activate pathways such as protein kinase C, leading to endothelial dysfunction and abnormal immune cell activation, thereby promoting vascular damage and contributing to microvascular complications (42, 43).

Meanwhile, a persistent hyperlipidemic state can also cause vascular endothelial dysfunction and drive the progression of atherosclerosis, while promoting fatty acid-binding protein 5-mediated pro-inflammatory responses in macrophages, exacerbating inflammation and tissue damage (70). Therefore, under the dual toxicity of hyperglycemia and hyperlipidemia, molecular pathways associated with the risk of cardiovascular diseases, tumors, and metabolic complications are more likely to be enriched compared to a hyperlipidemic state alone (44–46).

Compared to MASLD, the transcriptional networks in T2DM with MASLD mice showed greater enrichment in inflammation-related pathways. This suggests that T2DM with MASLD may have more significant features of chronic inflammatory activation compared to MASLD alone. Inflammation, insulin resistance, liver fibrosis, and the progression of systemic complications are closely interrelated and mutually reinforcing, potentially forming a crucial mechanistic basis for accelerated disease progression.

Transcriptomic analysis revealed significant downregulation of GYS2 in both T2DM with MASLD and MASLD-only mice. It is important to emphasize that GYS2 encodes glycogen synthase 2, a key rate-limiting enzyme for hepatic glycogen synthesis (47, 48). Under physiological conditions, insulin promotes glycogen synthesis-related processes and suppresses gluconeogenesis via the PI3K/AKT signaling pathway, thereby maintaining glucose homeostasis (49). When GYS2 expression or activity is reduced, the capacity for hepatic glycogen synthesis and storage —a critical postprandial nutrient disposal pathway— is impaired. This may weaken the postprandial glucose buffering capacity and divert substrates towards lipid synthesis pathways, thereby promoting hepatic lipid deposition, exacerbating insulin resistance, and forming a vicious metabolic cycle.

Previous population genetic and imaging studies also support the association between GYS2 and hepatic fat accumulation: a genetic association study suggested that GYS2 is associated with the liver proton density fat fraction, a quantitative fat measurement, indicating that the glycogen metabolism pathway may play an important role in hepatic fat deposition. Some genetic variants may participate in the development of MASLD through mechanisms affecting insulin sensitivity, among others (50). Additionally, glycogen storage disease type 0 serves as a classic clinical model of GYS2 dysfunction. Its core mechanism involves GYS2 gene mutations leading to significantly impaired hepatic glycogen synthesis capacity. Clinically, it can present with fasting hypoglycemia and postprandial hyperglycemia, and a high proportion of patients develop hepatic steatosis (51). This evidence is consistent with the observed downregulation of GYS2 in this study and the speculation that it may be involved in glucose and lipid metabolic reprogramming.

Moreover, our comparison revealed a more pronounced downregulation trend of hepatic GYS2 in T2DM with MASLD mice compared to MASLD-only mice. This suggests that GYS2-related glycogen synthesis impairment may be more prominent in the context of T2DM with MASLD, thereby associating with more severe metabolic disorders and hepatic pathological progression. However, the direct cause of GYS2 downregulation and its causal role in the progression of this comorbidity require further clarification. Combining the multi-omics correlation results from this study, the elevation of *α*-HB was significantly correlated with GYS2 downregulation, suggesting that α-HB may act as a key metabolic signal involved in regulating GYS2 and glycogen metabolism, thereby promoting the progression of the comorbidity.

Through metabolomic analysis, this study compared differential metabolites among normal control mice, T2DM with MASLD mice, and MASLD-only mice. It was found that compared to control mice, both T2DM with MASLD mice and MASLD mice exhibited significant phenotypes of lipid metabolism disorder. However, T2DM with MASLD mice, in addition to lipid metabolism abnormalities, also displayed more prominent glucose and energy metabolism disorders, particularly characterized by increased levels of ketone body-related metabolites. Previous research suggests that excessive fatty acid accumulation in the liver can provide substrates for ketogenesis (52). Concurrently, insulin is a key hormone inhibiting ketogenesis; insulin resistance or relative/absolute insulin deficiency can lift this inhibition, thereby promoting enhanced hepatic ketogenesis (53). Against the backdrop of glucose metabolism disorders, an imbalance between ketone body production, utilization, and clearance may collectively lead to abnormal ketone metabolism. Collectively, excessive fatty acid substrate, coupled with insulin resistance lifting ketogenesis suppression, likely explains the more pronounced ketone body and energy metabolism disturbances in the comorbid model.

Upon further screening of differential metabolites, we found that the level of *α*-HB was significantly elevated in T2DM with MASLD mice, suggesting its potential involvement in the metabolic regulation of the comorbid state. *α*-HB is increasingly recognized not merely as a byproduct but as a nutritional metabolite indicative of metabolic stress. *α*-HB can be produced in various metabolic bypass or conversion reactions, such as those related to amino acid catabolism (54, 55). Existing research has primarily focused on the potential value of *α*-HB in early prediction of diabetes and metabolic risk assessment, indicating its close association with metabolic disorders such as insulin resistance and oxidative stress (56, 57), and its potential as a biomarker (58–60). In this study, correlation analysis between metabolomics and transcriptomics further revealed that elevated *α*-HB levels were significantly associated with downregulated GYS2 expression. Based on this, we propose the hypothesis: α-HB, as a circulating nutritional stress signal, may impair insulin sensitivity and promote the progression of T2DM with MASLD by disrupting GYS2-mediated hepatic nutrient storage.

To validate the role of *α*-HB in T2DM with MASLD, this study administered *α*-HB via intraperitoneal injection to normal mice and T2DMwith MASLD mice, respectively. The results showed that compared to the non-intervention group, FBG and insulin levels were significantly increased in mice after *α*-HB intervention, suggesting a further decline in insulin sensitivity and aggravation of insulin resistance in α-HB-intervened T2DM with MASLD mice compared to their non-intervened counterparts. This result is consistent with previous evidence: for example, improved blood glucose in diabetic patients after gastric bypass surgery may be related to a significant postoperative decrease in plasma *α*-HB, and α-HB levels are positively correlated with the insulin resistance index (61). Regarding the potential mechanism by which *α*-HB promotes insulin resistance, studies have proposed that it may mediate metabolic imbalance through pathways such as elevating glutathione-related oxidative stress levels and enhancing lipid oxidation (56).

In addition to glucose metabolism, *α*-HB intervention also further exacerbated lipid metabolism disorders. Detection in this study showed that *α*-HB intervention significantly increased serum TG levels in T2DM with MASLD mice. Although differences in cholesterol-related indicators did not reach statistical significance, a downward trend in HDL-C and an upward trend in LDL-C were observed. Concurrently, quantitative analysis of liver Oil Red O staining suggested an increase in intrahepatic lipid droplet area and more pronounced lipid deposition after *α*-HB intervention, indicating aggravated steatosis. These results support that α-HB can promote lipid deposition and accelerate MASLD-related pathological progression. This underscores the role of *α*-HB in driving hepatic nutrient partitioning away from glycogen towards lipid. Previous reports, such as that by Russell P. Goodman et al., also indicated a correlation between α-HB and hepatic lipid metabolism disorder, although its specific mechanism remains unclear (62). The findings of this study provide further mechanistic clues for this association.

Regarding the inflammatory response, previous studies suggest that exogenous administration of *α*-HB can induce a pro-inflammatory response characterized by elevated IL-6 and TNF-α, thereby further impairing hepatic and systemic insulin sensitivity (63). Potential mechanisms include: α-HB triggering the translocation of lactate dehydrogenase A to the nucleus, prolonging nuclear retention of NF-κB, enhancing TNF-α production, and affecting insulin secretion (63). This study observed a consistent trend: after α-HB intervention, TNF-α and IL-6 were significantly elevated in the serum of both normal mice and T2DM with MASLD mice. Meanwhile, liver HE staining indicated more obvious inflammatory cell infiltration, suggesting that *α*-HB may further amplify the processes of insulin resistance and liver injury through “metabolism-inflammation” coupling.

To further verify whether α-HB affects hepatic GYS2 expression, this study conducted validation at three levels: semi-quantitative immunohistochemical analysis, Western blot, and QPCR. The results showed that GYS2 expression in the cytoplasm of hepatocytes was significantly downregulated in both α-HB-intervened normal mice and T2DM with MASLD mice compared to the non-intervention groups, with consistent downward trends at both mRNA and protein levels, suggesting that *α*-HB intervention may downregulate GYS2 expression. Furthermore, considering the important role of GSK3β in insulin resistance and glycogen synthesis regulation (55, 64), studies indicate that abnormal activation of GSK3β in an insulin-resistant environment may lead to inhibited GYS2 function and impaired hepatic glycogen synthesis (49). RT-qPCR in this study suggested upregulated GSK3β expression after *α*-HB intervention; Western blot results showed a decreased P-GSK3β (Ser9)/GSK3β ratio, indicating enhanced GSK3β activity. Combining this with research by the Irimia JM team indicating that GYS2 knockout enhances hepatic lipogenic capacity (65), and the results of this study showing GYS2 downregulation accompanied by reduced hepatic glycogen and increased hepatic TG, it is suggested that *α*-HB may, by activating GSK3β and downregulating/inhibiting the GYS2-related glycogen synthesis pathway, lead to restricted glycogen synthesis and aggravated lipid deposition, thereby exerting dual adverse effects on glucose and lipid metabolism and exacerbating hepatic insulin resistance.

Overall, this study supports that elevated α-HB may be one of the important metabolic features of T2DM with MASLD, serving as a critical node linking nutrient overload to hepatic metabolic dysfunction. It may promote hepatic lipid deposition, inflammatory activation, and aggravated insulin resistance through the mechanistic chain related to GSK3β activation—GYS2 downregulation—glycogen synthesis impairment, thereby driving disease progression. From a nutritional perspective, the *α*-HB–GSK3β–GYS2 axis can be viewed as a pathogenic pathway that translates a dysregulated nutritional milieu into defective hepatic energy partitioning, favoring lipid accumulation over glycogen synthesis. Therefore, *α*-HB and its downstream GSK3β/GYS2 axis may provide new research directions for risk stratification, biomarker panel construction, and potential intervention targets for T2DM with MASLD.

## 5 Limitations

This study has certain limitations that warrant further refinement. Although a decrease in GYS2 expression was observed following *α*-HB intervention, the direct molecular mechanism by which α-HB influences GYS2 remains unclear. Although our study provides multi-level evidence for GSK3β activation, direct measurement of GYS2 phosphorylation at the specific inhibitory sites was not performed. This remains a technically important validation for future, more targeted mechanistic studies on the post-translational regulation of this pathway. Previous studies suggest that elevated TNF-α and IL-6 may indirectly affect GYS2 expression and activity by activating pathways such as JNK and SOCS3 (66–68). Therefore, future research could focus on validating mechanisms related to inflammatory signaling, insulin pathways, and GSK3β-mediated phosphorylation regulation to clarify the key steps in *α*-HB’s action. Given that GYS2 is specifically expressed in the liver, the present study was designed to elucidate its associated hepatic mechanisms. Consequently, the potential systemic effects of α-HB on skeletal muscle metabolism were not explored and represent a valuable direction for future research. Furthermore, the high *α*-HB state in this study was primarily achieved through short-term, relatively high-dose intraperitoneal injection, whereas clinical α-HB elevation is often chronic, and the effects of acute intervention may differ from those of chronic exposure. Future studies could extend the intervention duration, optimize the administration method, or employ models that more closely mimic physiological conditions to validate the generalizability of the findings. Finally, the current findings are primarily based on correlations and phenotypic support; the next step will involve conducting mechanistic rescue experiments focusing on the “α-HB–GSK3β/GYS2” pathway, employing strategies such as GYS2 overexpression/knockdown to further validate the dependency of glycogen synthesis impairment and lipid deposition phenotypes on α-HB.

## 6 Conclusion

This study employed a high-fat diet combined with low-dose STZ to establish a T2DM with MASLD model. Utilizing metabolomics and transcriptomics technologies, it systematically delineated the metabolite profiles, transcriptomic differences, and enriched pathway characteristics of the comorbid state compared to normal controls and MASLD alone, identifying α-HB as a key differential metabolite. Furthermore, multi-omics correlation analysis revealed a significant association between elevated α-HB and downregulated GYS2. In animal intervention experiments, exogenous α-HB exacerbated hyperglycemia and insulin resistance, promoted serum lipid disorders and hepatic lipid deposition, enhanced pro-inflammatory cytokine levels and hepatic tissue inflammatory response, while being accompanied by decreased hepatic GYS2 expression and reduced glycogen. These findings suggest that α-HB may participate in the progression of T2DM with MASLD by affecting GSK3β/GYS2-related glycogen metabolism processes. The aforementioned discoveries provide new research clues and insights for biomarker screening and the exploration of potential intervention targets for T2DM with MASLD.

## Data Availability

The datasets presented in this study can be found in online repositories. The names of the repository/repositories and accession number(s) can be found at: https://www.ncbi.nlm.nih.gov/, PRJNA1368300; https://www.ebi.ac.uk/metabolights/, MTBLS13389.
