# Supplementary material for: Glycogen metabolic dysfunction in T2DM with MASLD: linking α-hydroxybutyrate to GYS2 downregulation
Source: Front Nutr. 2026 Jun 22;13:1860017. doi: 10.3389/fnut.2026.1860017 (PMC13334271; doi:10.3389/fnut.2026.1860017)
Supplement: Supplementary file 1 [file Data_Sheet_1.PDF]

Abbreviations

DM	Diabetes mellitus
GYS2	Glycogen synthase 2
GSK3β	Glycogen synthase kinase 3 β
H&E	Hematoxylin and Eosin Staining
HDL-C	High density lipoprotein cholesterol
IL-6	Interleukin-6
KEGG	Kyoto Encyclopedia of Genes and Genomes
LDL-C	Low density lipoprotein cholesterol
MASLD	Metabolic associated steatotic Liver disease
RNA-seq	RNA sequencing
RT-qPCR	Real-Time Quantitative Polymerase Chain Reaction
STZ	Streptozotocin
TG	Triglyceride
TNF-α	Tumor necrosis factor-α
WB	Western blot
α-HB	α-hydroxybutyrate
