# Supplementary figures and images for: Glycogen metabolic dysfunction in T2DM with MASLD: linking α-hydroxybutyrate to GYS2 downregulation
Source: Front Nutr. 2026 Jun 22;13:1860017. doi: 10.3389/fnut.2026.1860017 (PMC13334271; doi:10.3389/fnut.2026.1860017)

GYS2 ( $\beta$ actin)

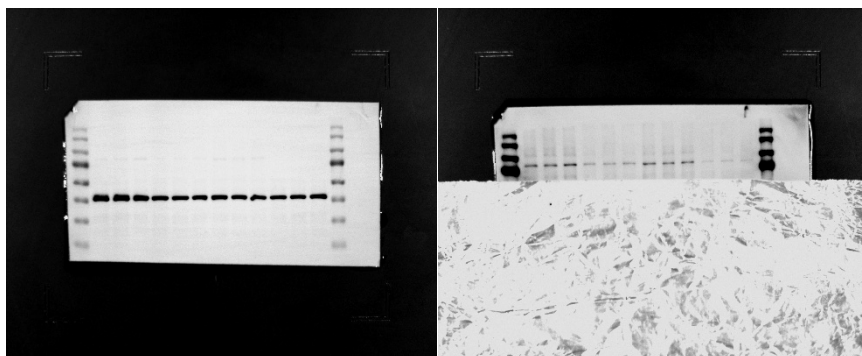

GSK3 $\beta$ (GAPDH)

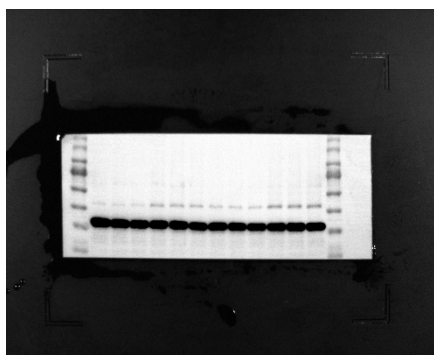

P-GSK3 $\beta$ (GAPDH)

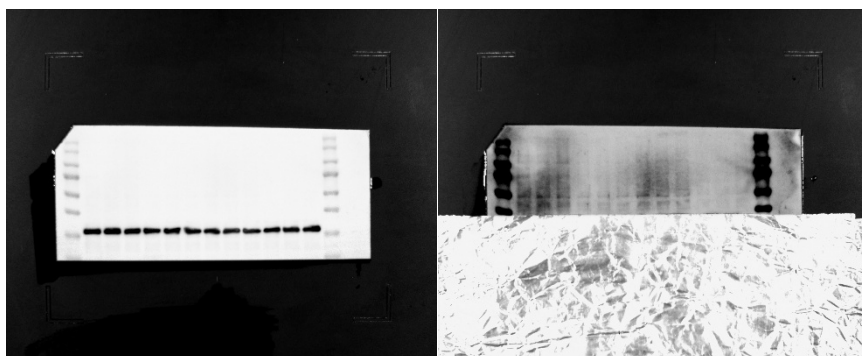

Supplement: Supplementary file 2 [file Image_1.PDF]
